# Brain metastases

**DOI:** 10.3332/ecancer.2012.ed15

**Published:** 2012-10-17

**Authors:** Nick Plowman

**Affiliations:** St Bartholomew’s Hospital, London, UK

The spread of cancer to the brain is becoming a more common problem in Clinical Oncology and up to 40% of cancer patients suffer from this form of disease. There are two reasons: the first is that the control of primary tumours is improved and so the patients are living longer, during which time the cancer has time to spread to unusual sites such as the brain; secondly, there is a barrier to large hydrophilic molecules (which applies to the majority of cytotoxic drugs) that prevents their penetration into the brain (the so-called blood-brain barrier). I give an example: HER-2 positive breast cancer, comprising approximately 20% of all breast cancers, has a higher likelihood to spread to brain than other types of this cancer; however, the specific monoclonal antibody that has made such an important impact on the improved survival of these patients viz. trastuzumab/herceptin, does not penetrate the blood-brain barrier. The consequence has been a higher than previous rate of brain metastases in this disease – the patients do not relapse so frequently in the body (systemically) due to the herceptin, but relapse more in the brain. There are other examples in other diseases. All this has focussed the minds of neuro-oncologists on optimising the treatment of these brain metastatic disease patients.

The most common cancers to spread to the brain are lung cancer (the particularly aggressive small cell lung cancer in particular but also non-small cell types), breast cancer (and we have noted the particular problem of HER-2 positive disease), renal, gastrointestinal cancers and melanoma. In small cell lung cancer, the risk of brain relapse is so high that, after chemotherapy induced remission of the systemic disease (by drugs that do not easily penetrate the brain), it is now common practice to deliver prophylactic brain radiotherapy to pre-emptively strike at the microscopic disease that would otherwise later manifest (and be later more difficult to control).

The presentation of the patient with brain metastases may be in various ways. The patient may present with an epileptic seizure (as any abnormal growth in the brain acts as an irritable focus) or with a neurological deficit (e.g the weakness of a limb if the metastasis is in the motor cortex) or with headaches and drowsiness if the tumour is so large as to raise the intracranial pressure.

Brain metastases may be single or multiple. With the advent of high quality MRI scanning of the brain, it is less common to find solitary brain metastases. This statement is particularly true for lung and breast cancer (which have a particular predisposition to multiple brain metastases), but it is a first priority to discern as the optimal therapy differs.

Once the brain scan has been performed and the diagnosis made (and for a solitary metastasis, the differential diagnosis from a primary brain tumour needs to be distinguished) then the clinician needs to consider the therapy options:

In the patient who is in systemic remission from the cancer and fit from other health viewpoints, and who has a moderate to large metastasis that is superficially placed in the brain and causing pressure here, there is no doubt that primary surgical removal/debulking is the best first therapy. This should be followed by wide field brain radiotherapy as there are considerable data to show that surgery alone is attended by a higher relapse in the brain than in patients receiving post operative radiotherapy. Thus, in the 1987 Mayo Clinic series, the risk of subsequent relapse in the brain was reduced from 85% to 21% by the addition of post operative radiotherapy. However, radiotherapy alone is not a substitute for surgery where there are sizeable superficial metastasis/es.

So, standard practice has been surgery (where appropriate) followed by wide-field/whole brain radiotherapy and the usual radiotherapy dose prescription has been one of 30 Gy in 10 daily fractions using megavoltage photons (range: 20 Gy in 5 fractions to 40 GY in 15–20 fractions). The treatment is well tolerated, except for hair loss. Most patients respond, but not all durably.

The survival of such patients depends on several important factors: patients aged below 65 years, with a controlled primary cancer and no systemic metastases, who had a good performance status, and only one brain metastasis ( on good quality MRI scan – and that one tumour not of threatening size and inoperable) had the best survival, whereas those patients with any of the above factors in reverse (i.e. old age, poor performance status, systemic metastases, and multiple brain metastases) had a survival of at most a few months. The histology of the primary cancer has some influence too – both because some histologies of cancer respond better to radiation and secondly because some cancers tend to ‘throw up’ multiple brain metastases, with the knock on effect on prognosis.

Then came along stereotactic radiation therapy (nickname: radiosurgery) – the ability to ‘focus’ (or anyway concentrate) a very high dose of radiation on an brain metastasis, sparing the surrounding brain because the high dose zone of radiation has a steep dose gradient that ‘falls away’ at the margin of the MR mapped target lesion. Both Gamma Knife (Elekta, Linkoping, Sweden) and Cyberknife (Accuray, California) are examples of this technology and have brought a powerful new methodology for treating individual brain metastases – both up front (unirradiated brains) and in the relapse situation (in patients who have already received wide field brain radiotherapy).

A randomised clinical trial published in the Lancet in 2004 demonstrated that for patients with one (and probably up to three) brain metastases, the addition of a stereotactic radiosurgery to whole brain radiotherapy led to improved survival, most significant in good performance status patients. For patients with single (or a few) brain metastases, where surgery was not appropriate (e.g. deep seated growths), this has led to a huge new group of patients who are helped by this technology. I usually give the wide-field brain radiotherapy and then scan a month later and give the boost to any lesion that has survived.

The rationale for this success is that established (sizeable) brain metastases are more difficult for the wide-field brain radiotherapy to sterilise and often early shrinkage turns out to be temporary and is later followed by regrowth

The technology is also useful in those patients who have had whole brain radiotherapy and yet relapse in one or two sites later (and remain in good shape from the systemic cancer viewpoint) for they cannot safely have wide- field radiotherapy again.

Meanwhile, the development of drugs that penetrate the blood-brain barrier has increased the interest in systemic therapies to treat brain metastatic disease, not least because the barrier is partially deficient in many metastases. Lapatinib – a HER-(1+02 inhibitor and a small molecule compared with herceptin has some activity in HER-2 positive breast cancer patients with brain metastases; this type of advance may have further usefulness in the future.

